# Prevalence and risk factors of early postoperative seizures in patients with glioma: a systematic review and meta-analysis

**DOI:** 10.3389/fneur.2024.1356715

**Published:** 2024-03-20

**Authors:** Bo Sun, Yuchen Sun, Zijian Wang, Chao Zhao, Liang Yang

**Affiliations:** Department of Neurosurgery, The Second Hospital of Hebei Medical University, Shijiazhuang, China

**Keywords:** glioma, seizures, risk factors, meta-analysis, systematic review

## Abstract

**Objective:**

This study aimed to explore the prevalence and risk factors of early postoperative seizures in patients with glioma through meta-analysis.

**Methods:**

Case–control studies and cohort studies on the prevalence and risk factors of early postoperative seizures in glioma patients were retrieved from various databases including CNKI, Wanfang, VIP, PubMed, Embase, Cochrane Library, and Web of Science, and the retrieval deadline for the data was 1 April 2023. Stata15.0 was used to analyze the data.

**Results:**

This review included 11 studies consisting of 488 patients with early postoperative seizures and 2,051 patients without early postoperative seizures. The research findings suggest that the prevalence of glioma is complicated by seizures (ES = 19%, 95% confidence interval [CI] [14%−25%]). The results also indicated a history of seizures (RR = 1.94, 95% CI [1.76, 2.14], *P* = 0.001), preoperative dyskinesia (RR = 3.13, 95% CI [1.20, 8.15], *P* = 0.02), frontal lobe tumor (RR = 1.45, 95% CI [1.16, 1.83], *P* = 0.001), pathological grade ≤2 (RR = 1.74, 95% CI [1.13, 2.67], *P* = 0.012), tumor≥ 3 cm (RR = 1.70, 95% CI [1.18, 2.45], *P* = 0.005), tumor resection (RR = 1.60, 95% CI [1.36, 1.88], *P* = 0.001), tumor edema ≥ 2 cm (RR = 1.77, 95% CI [1.40, 2.25], *P* = 0.001), and glioma cavity hemorrhage (RR=3.15, 95% CI [1.85, 5.37], *P* = 0.001). The multivariate analysis results showed that a history of seizures, dyskinesia, tumor ≥3 cm, peritumoral edema ≥2 cm, and glioma cavity hemorrhage were indicated as risk factors for glioma complicated with early postoperative seizures.

**Significance:**

Based on the existing evidence, seizure history, dyskinesia, frontal lobe tumor, pathological grade ≤2, tumor ≥3 cm, partial tumor resection, edema around tumor ≥2 cm, and glioma cavity hemorrhage are indicated as risk factors for glioma complicated with early postoperative seizures.

## 1 Background

Gliomas originate from the abnormal proliferation of glial cells in the brain (1, 2), and they are common primary central nervous system tumors (3), with a high mortality risk (4). The annual occurrence of gliomas is about 5–8 cases per 100,000 people, while the annual mortality rate is as high as 30,000, ranking third after pancreatic and lung cancers (5–7). In patients with gliomas, seizures are often the initial symptom and the reason for seeking medical attention. Glioma-related seizures are typical accompanying symptoms of gliomas, especially in low-grade gliomas (8, 9), where the main symptoms are sudden paroxysmal convulsions, foaming at the mouth, loss of consciousness, and convulsions of the limbs (10). The specific mechanisms underlying glioma-related seizures are not yet fully understood, and pathological changes and pathogenic mechanisms are diverse, likely resulting from the combined effects of multiple factors (11, 12). The abnormal expression of tumor-related genes is considered to be closely associated with the occurrence and development of seizures in brain tumors, especially gliomas (13). Although advances in surgery and chemoradiotherapy have improved the prognosis of glioma cases, the presence of seizures severely affects the quality of life for patients (14). Currently, the clinical efficacy of treating glioma-related seizures remains unsatisfactory because of an incomplete understanding of the pathophysiological, biochemical, molecular, and pharmacological mechanisms underlying its occurrence (15, 16). Giraldi et al. (17) found that there exist an obviously increased cumulative risk of new-onset seizures after craniotomy. Previous studies confirmed that seizures are a common complication of intracranial gliomas that can lead to disability or even death. Therefore, it is important to determine the relevant risk factors for early postoperative seizures in gliomas cases. However, the risk factors associated with gliomas remain controversial (18). Therefore, this study aimed to address these controversies through a meta-analysis investigating such risk factors. It is hoped that, by preventing and treating early postoperative seizures, the quality of life and outcomes of glioma patients can be improved.

## 2 Materials and methods

This systematic review is conducted according to the preferred reporting items of the Protocol for Systematic Reviews and Meta-analyses (PRISMA-P) guidelines. The review will be conducted according to PRISMA criteria (19). The registration number is CRD42023415658.

### 2.1 Literature search

We searched the CNKI, Wanfang, VIP, PubMed, Embase, Cochrane Library, Web of Science, and other databases to identify the risk factors of seizures in glioma cases. The search deadline for the data was 1 April 2023. Subjects and free words were used for retrieval: seizures, glioma, and risk factors. See Supplementary material 1 for specific retrieval strategies.

### 2.2 Inclusion and exclusion criteria

The study included adults who met the diagnostic criteria for glioma (including low- and high-grade gliomas) (20), and the exposure factor was early postoperative seizures (occurred within 7 days after surgery). The primary outcome was the prevalence of postoperative seizures, while the secondary outcome was risk factors for postoperative seizures. The eligible study designs included case–control and cohort studies.

The exclusion criteria encompassed meta-analyses, protocols, letters, repeatedly published articles, systematic reviews, failure to obtain full text, failure to obtain available data, and animal experiments.

### 2.3 Data extraction

Two independent evaluators performed the literature screening to extract data and directly screened the easily judged literature by reading the titles, abstracts, and the full text. Any disagreements were resolved by consulting relevant experts. The selection criteria were strictly followed during the screening process. They extracted the corresponding indicators from the studies and cross-checked the extracted data to ensure consistency. The primary collected data included the name of the first author, year of publication, country, study design, sample size, sex, and age.

### 2.4 Quality evaluation

The Newcastle-Ottawa Scale (NOS) (21) was utilized to assess the case–control studies, including the study population (4 points) and measurement of exposure factors or results (3 points). The total score of the scale is 9, with scores of ≤ 4, 5–6, and ≥7 indicating low, medium, high quality, respectively. If the two researchers disagree on the evaluation process, they will discuss the decision or seek input from a third party.

### 2.5 Statistical analysis

Stata 15.0 was used to statistically analyze the data. Risk values for each study were expressed as RR values, with corresponding 95% confidence intervals (CIs) calculated. The heterogeneity test (Q test) and I^2^ statistics were used to select the appropriate model for calculating the pooled RR. If I^2^ > 50%, the random-effects model was adopted; if I^2^ ≤ 50%, the fixed-effects model was applied. For I^2^ > 50%, we assessed the sensitivity of the literature using the leave-one-out method. Additionally, we assessed publication bias using the Egger test, with a significance level set at 0.05.

## 3 Results

### 3.1 Results of the literature retrieval

**Figure 1:**
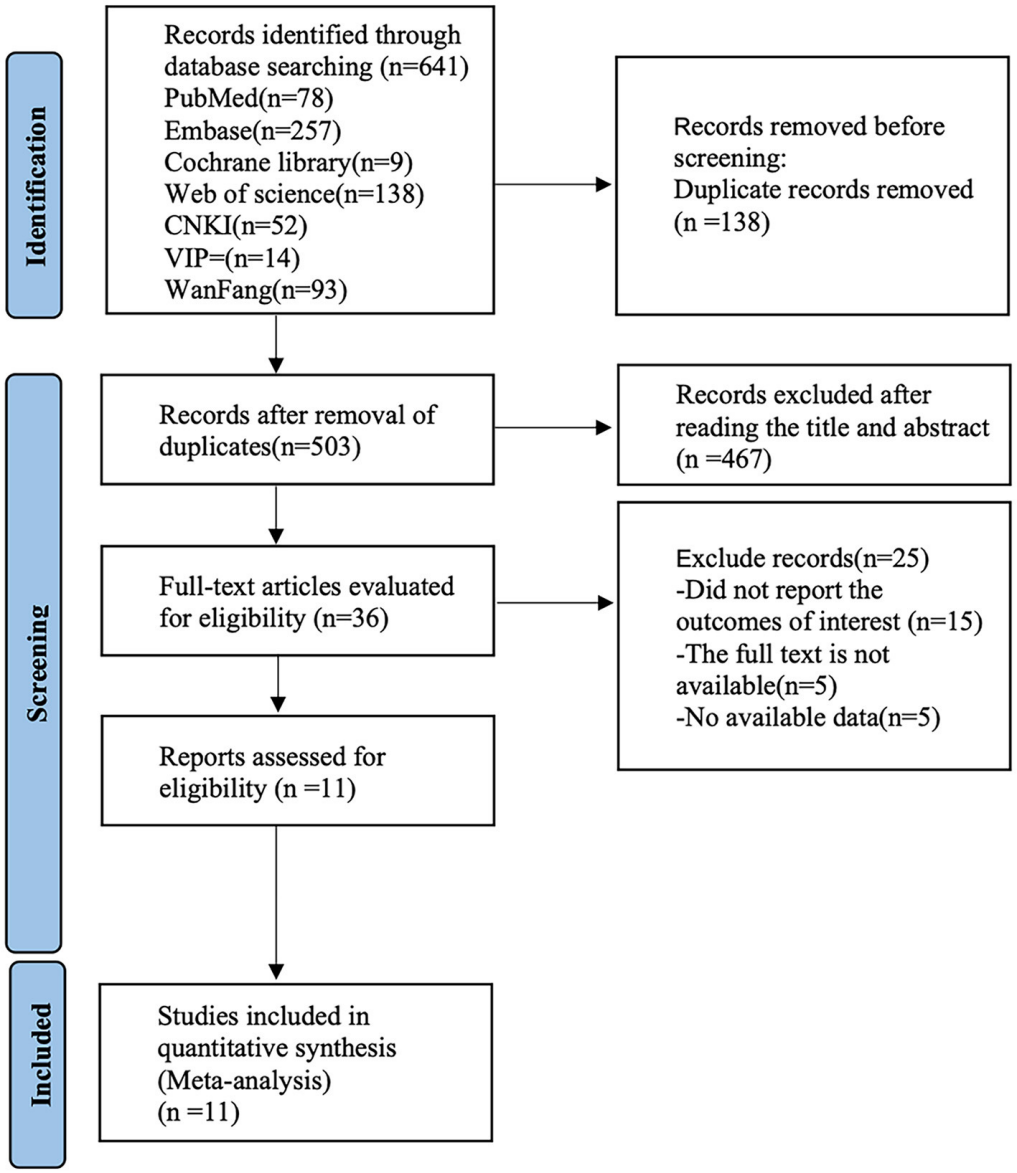
Flow chart of the literature search.

By searching the CNKI, Wanfang, VIP, PubMed, Embase, Cochrane Library, and Web of Science databases, 641 documents were initially obtained, 503 documents were retained after removing duplicate documents, 36 articles were preliminarily screened by reading the titles and abstracts, and 11 documents were included after reading the full text. Figure 1 shows the retrieval flowchart.

### 3.2 Basic features of the included literature

The 11 included (22–32) studies were case–control studies, involving 488 patients with early postoperative seizures and 2,051 patients without early postoperative seizures. The inclusion criteria specified that the age of patients ranged from 7 to 81 years. See Supplementary Table S1 for specific document characteristics. Eleven articles were evaluated using the NOS criteria: one (23) scored 6 points, indicating that the research quality was moderate. The remaining articles scored 7–8 points, and the overall quality of those articles was high. See Supplementary Table S2 for specific quality evaluations.

### 3.3 Prevalence of glioma with early postoperative seizures

Eleven studies (22–32) have reported the prevalence of gliomas complicated by early postoperative seizures. The heterogeneity test (I^2^ = 92.3%, *P* = 0.001) was conducted based on the random-effects model. It was found that the prevalence of glioma is complicated by early postoperative seizures (ES = 19%, 95% CI [14%−25%]) because the heterogeneity of the indicators was large; therefore, sensitivity analysis was carried out to eliminate them one by one. The analysis results indicated that the sensitivity was low, and the analysis results were stable. The Egger test was performed on the index to evaluate publication bias (*P* = 0.026), and the prevalence was more likely to indicate publication bias (see Supplementary Figure 1).

### 3.4 Single-factor meta-analysis

#### 3.4.1 Seizure history

**Figure 2:**
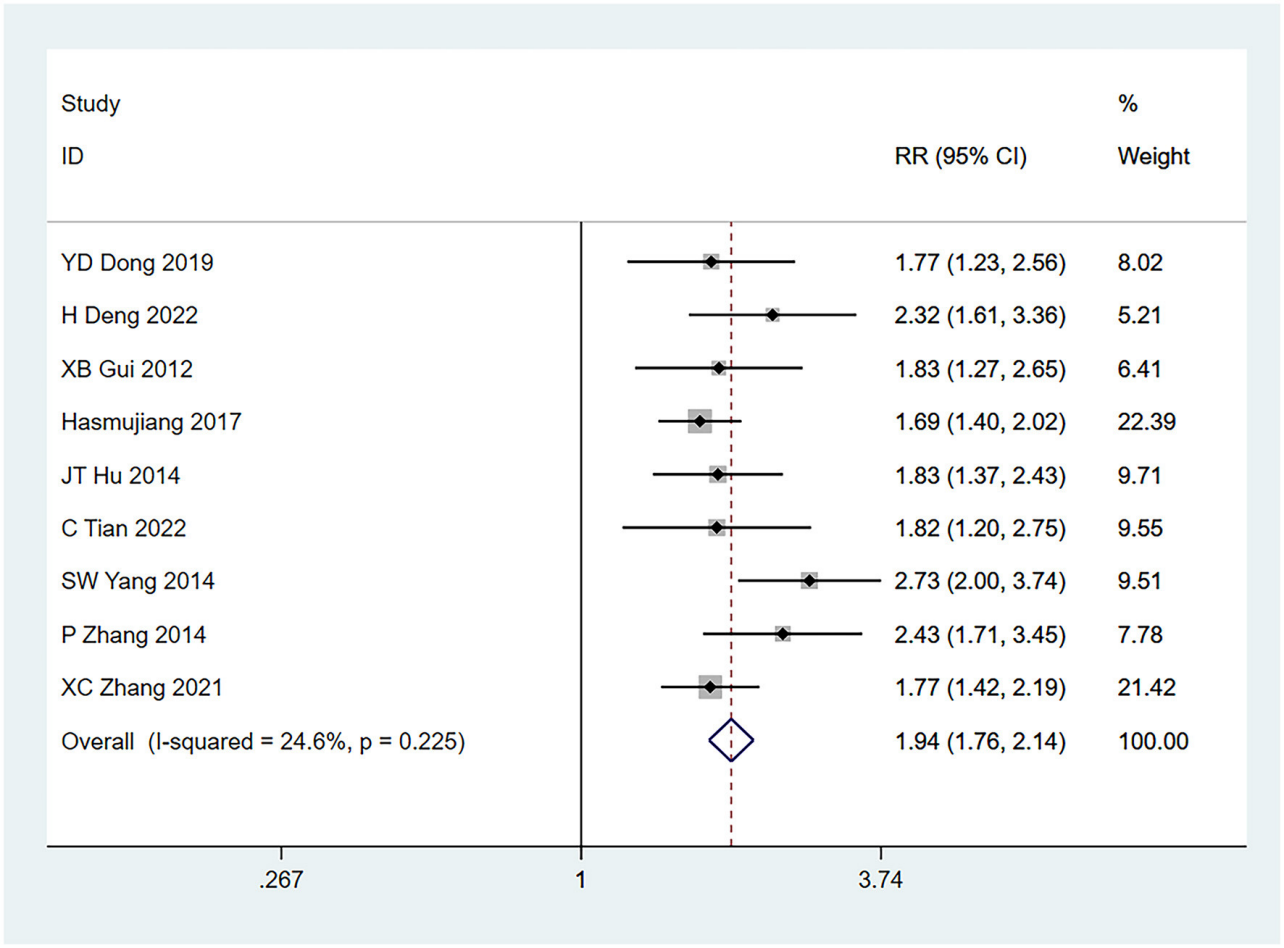
Forest plot of the meta-analysis of seizure history.

Nine studies (22–30) mentioned a history of seizures (defined as tumor-related seizures before surgery) as a risk factor for seizures, and the heterogeneity test (I^2^ = 24.6%, *P* = 0.225) was conducted based on the fixed-effects model. The results suggested that a history of seizures was a risk factor for glioma complicated by seizures, and there exists an obvious difference (RR = 1.94, 95% CI [1.76, 2.14], *P* = 0.001) (see Figure 2 and Supplementary Table S3).

#### 3.4.2 Preoperative dyskinesia

**Figure 3:**
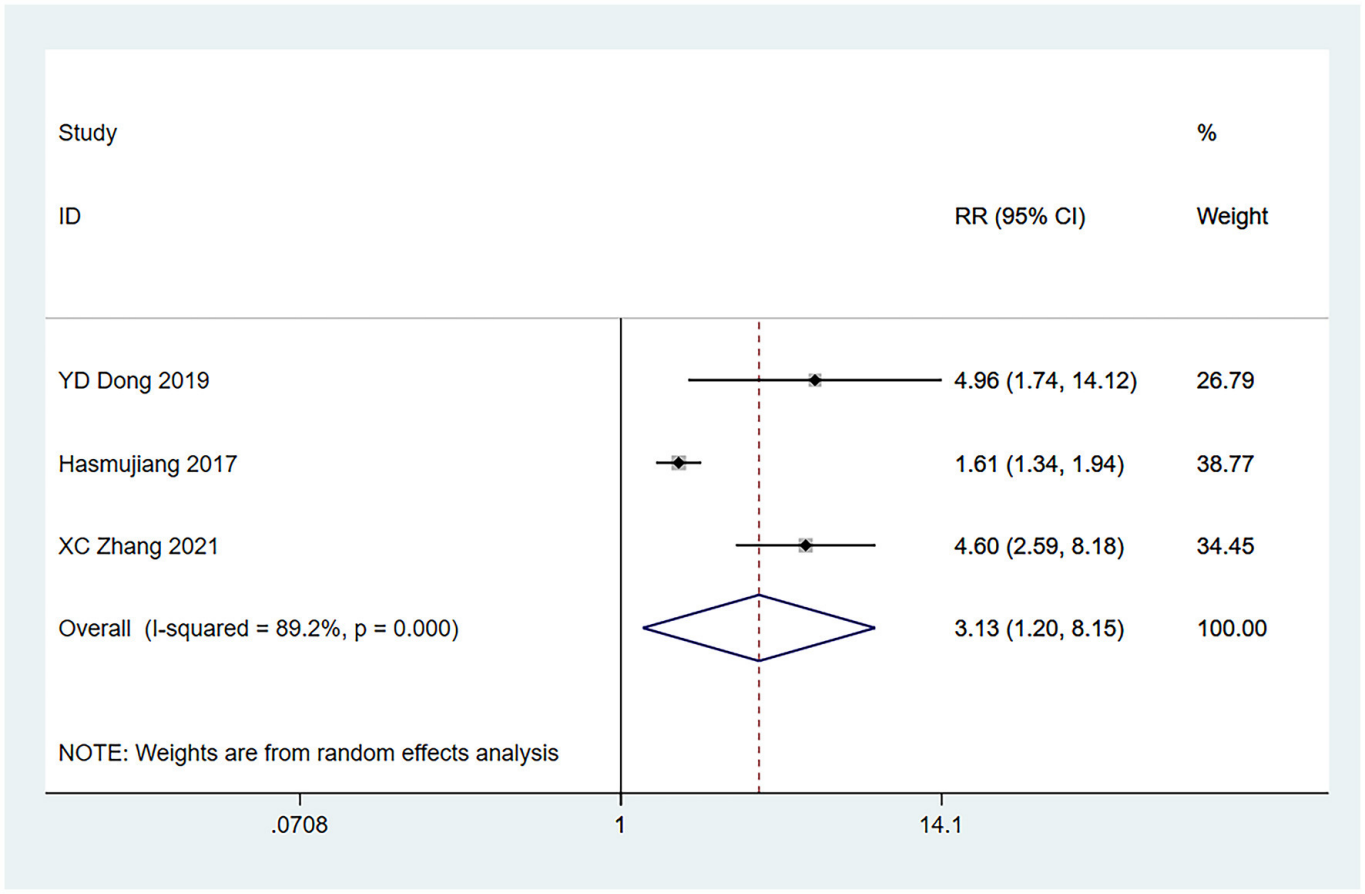
Forest plot of the meta-analysis of preoperative dyskinesia.

Preoperative dyskinesia (22, 25, 30) was mentioned as a risk factor in three studies, and the heterogeneity test (I^2^ = 89.2%, *P* = 0.001) was performed based on the random-effects model. It was found that preoperative dyskinesia was a risk factor for glioma complicated by seizures, and there exists an obvious difference (RR = 3.13, 95% CI [1.20, 8.15], *P* = 0.02) (see Figure 3 and Supplementary Table S3).

#### 3.4.3 Frontal tumor

**Figure 4:**
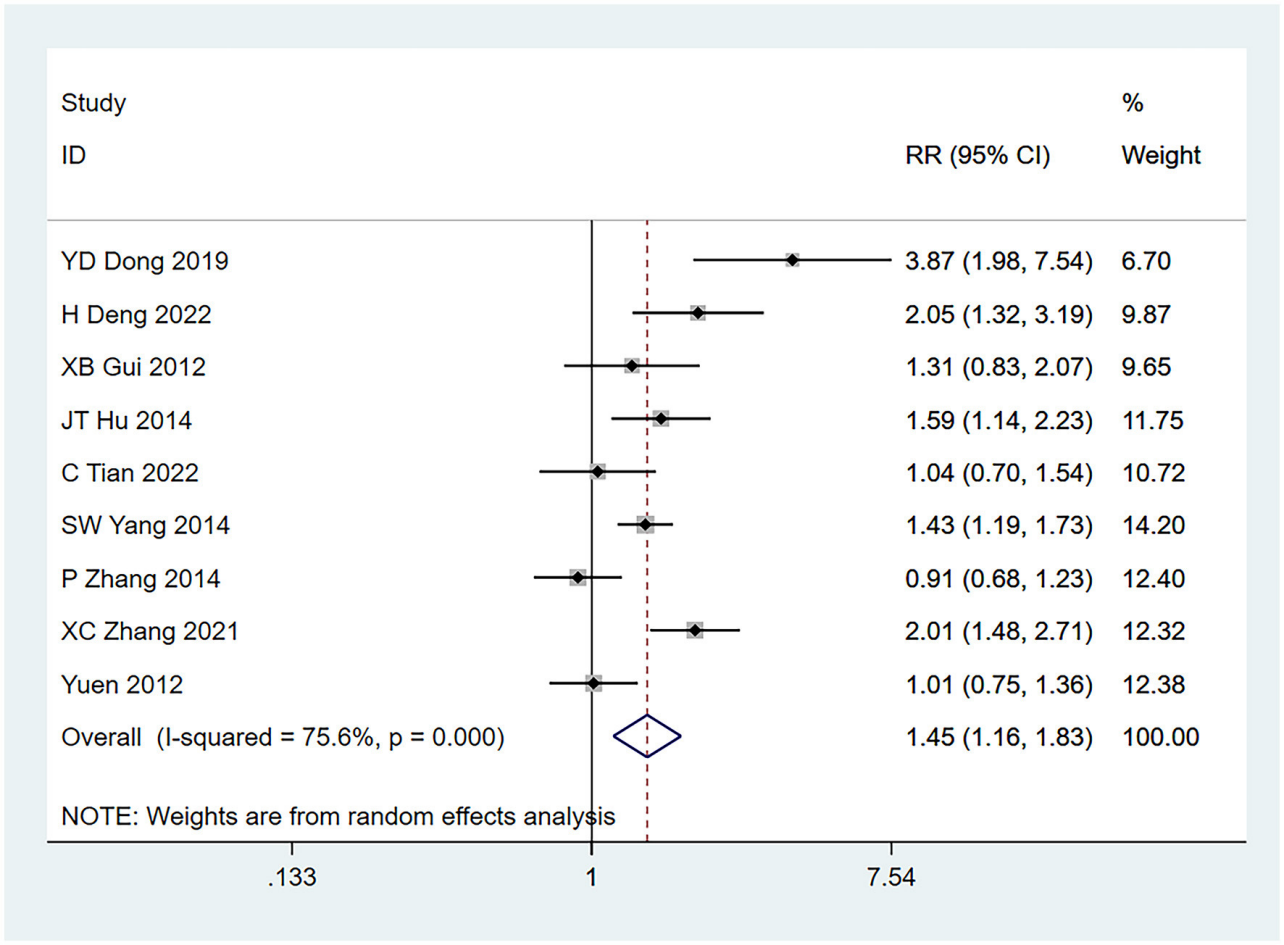
Forest plot of the meta-analysis of frontal lobe tumors.

Nine studies (22–24, 26–31) identified frontal lobe tumor location as a risk factor for seizures, and the heterogeneity test (I^2^ = 75.6%, *P* = 0.001) was performed based on the random-effects model. It was found that frontal lobe tumors were risk factors for glioma complicated with seizures, and there exists an obvious difference (RR = 1.45, 95% CI [1.16, 1.83], *P* = 0.001), as shown in Figure 4 and Supplementary Table S3.

#### 3.4.4 Pathological grade ≤2

**Figure 5:**
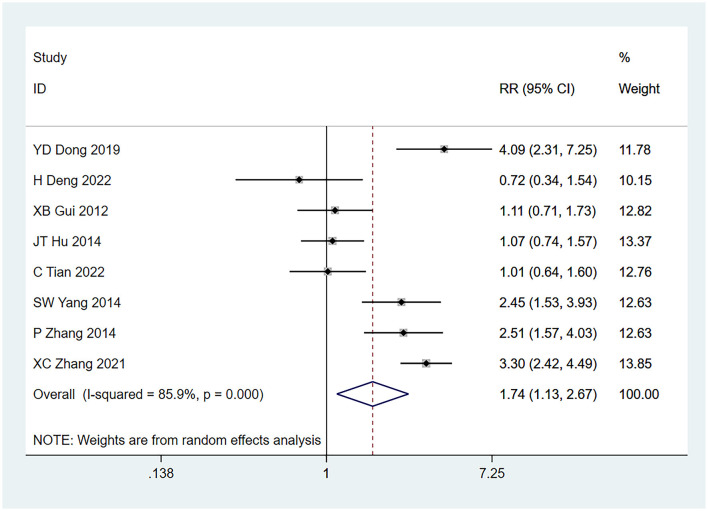
Forest plot of the meta-analysis of pathological grade <2.

Eight studies (22–24, 26–30) identified pathological grade ≤ 2 as a risk factor for seizures, and the heterogeneity test (I^2^ = 85.9%, *P* = 0.001) was performed based on the random-effects model. The results of the analysis reflected that pathological grade ≤ 2 was a risk factor for tumor-associated seizures and there exist an obvious difference (RR = 1.74, 95% CI [1.13, 2.67], *P* = 0.012) (see Figure 5 and Supplementary Table S3).

#### 3.4.5 Tumor ≥3 cm

**Figure 6:**
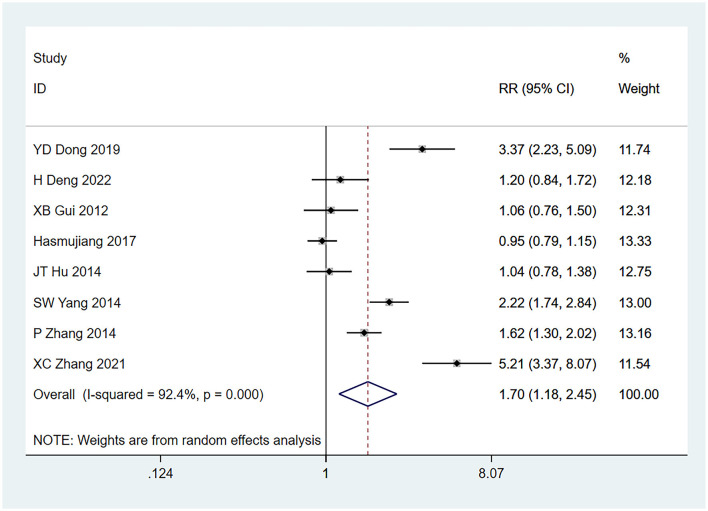
Forest plot of the meta-analysis of tumor >3 cm.

Eight studies (22–24, 26–30) mentioned tumors ≥3 cm as a risk factor for seizures, and the heterogeneity test (I^2^ = 92.4%, *P* = 0.001) was performed based on the random-effects model. It was found that tumors ≥3 cm were considered a statistically significant risk factor for tumor-associated seizures (RR = 1.70, 95% CI [1.18, 2.45], *P* = 0.005) (see Figure 6 and Supplementary Table S3).

#### 3.4.6 Partial resection of tumor

Seven studies (22–24, 26–29) mentioned partial tumor resection as a risk factor, and the heterogeneity test (I^2^ = 7.7%, *P* = 0.369) was performed based on the fixed-effects model. It was found that partial tumor resection was a statistically significant risk factor for tumor-associated seizures (RR = 1.60, 95% CI [1.36, 1.88], *P* = 0.001) (see Supplementary Table S3).

#### 3.4.7 Peritumor edema ≥2 cm

Six studies (22, 23, 25, 27–29) mentioned peritumor edema ≥2 cm as a risk factor for seizures, and the heterogeneity test (I^2^ = 63.7%, *P* = 0.017) was performed based on the random-effects model. As a result, peritumor edema ≥2 cm was found to be a statistically significant risk factor for tumor-associated seizures (RR = 1.77, 95% CI [1.40, 2.25], *P* = 0.001) (see Supplementary Table S3).

#### 3.4.8 Intracavitary hemorrhage of glioma

Six studies (22, 23, 25, 27–29) mentioned glioma cavity hemorrhage as a risk factor for seizures, and the heterogeneity test (I^2^ = 83.9%, *P* = 0.001) was conducted based on the random-effects model. It was found that glioma cavity hemorrhage was a risk factor for tumor-associated seizures, with obvious difference (RR = 3.15, 95% CI [1.85, 5.37], *P* = 0.001) (see Supplementary Table S3).

#### 3.4.9 Other meta-analysis results

Differences in the correlations between male and female sex, circulatory disease, metabolic disease, blurred tumor boundaries, prophylactic medication, and glioma with seizures were not statistically significant (Supplementary Table S3).

#### 3.4.10 Multi-factor meta-analysis results

The results of the multifactorial analysis mentioned in the research study were analyzed and combined. It was found that there exists an obvious difference in the correlation between frontal lobe tumors, partial tumor resection, prophylactic medication, and tumor-associated seizures. A history of seizures (ES = 2.54, 95% CI [1.24, 5.20], *P* = 0.011), motor impairment (ES = 2.53, 95% CI (1.83, 3.51), *P* = 0.001], tumor ≥3 cm (ES = 2.56, 95% CI [1.99, 3.31], *P* = 0.001), peritumor edema ≥2 cm (ES = 2.53, 95% CI [1.83, 3.51], *P* = 0.001), and glioma cavity hemorrhage (ES = 2.93, 95% CI (1.79, 4.81), *P* = 0.001) can be regarded as risk factors for tumor-associated seizures (see Supplementary Table S4).

## 4 Publication bias

The publication bias was assessed based on Egger's test for each risk factor. The *p*-values for each indicator, whether in univariate or multifactorial analysis, were >0.05, indicating no publication bias (see Supplementary Tables S3, S4).

## 5 Discussion

Many recent reports have used the review and meta-analysis to report the prevalence of seizures in patients with glioma. However, Zhang et al. (33) focused their study on assessing the relationship between tumor location and preoperative seizures in patients with glioma, Shan et al. (34) studied the prognostic factors of postoperative glioma and Audrey et al. (35) focused on the incidence of seizures in different glioma types. To the best of our knowledge, this study is the first to explore the risk factors of gliomas complicated by early postoperative seizures using a meta-analysis, the inclusion criteria for the current study differed from those used in all of the previous studies. Through the analysis of single and multiple factors, this study found that a history of seizures is a key risk factor of glioma complicated by seizures, indicating that the risk of seizures in glioma cases with preoperative seizures increased significantly. This may be related to the instability of neuronal membrane potential, decrease in seizure threshold, and abnormal discharge in patients with seizures, which is caused by abnormal and excessive discharge of cortical neurons. Therefore, patients with preoperative seizures should actively use drugs to reduce its occurrence (36). The present study also found an obviously higher occurrence of seizures in patients with dyskinesia in cranial glioma. However, previous literature found that there was no strong correlation between early postoperative epilepsy and dyskinesia (37). This conclusion may be due to the limited number of preoperative dyskinesia cases included in the present research; however, this highlights the need for future studies to pay closer attention to this aspect. Patients with preoperative dyskinesia, which indicates that their neurological function has been damaged to some extent and that they are more prone to epilepsy due to their inability to perform movements, need to be given priority attention to prevent epilepsy. The occurrence of seizures is significantly higher in cases with peri-tumoral edema ≥2 cm. A possible reason for this is that peritumoral edema includes both vasogenic and cytotoxic brain edema. Vasogenic brain edema increases capillary permeability and broken blood–brain barrier, leading to an abnormal distribution of ions inside and outside the cells, which in turn stimulates nerves and triggers seizures (38). In addition, when astrocytes are involved in cytotoxic brain edema due to increased occupancy effects, their ability to take up glutamate is reduced, which in turn affects the stability of the nerve cell membrane, inducing neuroexcitability and ultimately leading to seizures (39). This study also found that intratumoral hemorrhage is an independent risk factor for gliomas complicated by seizures. Intratumoral hemorrhage can cause brain tissue hypoxia and energy metabolism disorders. Iron ions in the blood can catalyze the production of oxygen free radicals, form lipid peroxides, and cause neuronal necrosis, leading to seizures. Therefore, intratumoral hemorrhage is considered a cause of early seizures (40). It has been shown that the tumor pathological grade is usually negatively correlated with early postoperative seizure events, possibly because seizures are characterized by abnormal neuronal firing. As the pathological grade of the glioma increases, infiltrative growth tends to become more pronounced, and this infiltrative growth of tumor cells may destroy projection fibers and neurons, thereby inhibiting the spread of seizure firing (41). This is in line with the previous study, which indicated that a pathological grade of ≤ 2 is an independent risk factor for tumor-associated seizures. For cases with larger tumors, the tumor is more likely to compress adjacent normal brain tissues, causing ischemia and metabolic disorders in the brain. This compression can result in an imbalance of intra- and extra-cellular ion levels, increasing the risk of seizures, which further supports the findings of the present study, indicating that tumors ≥3 cm are an independent risk factor for tumor-associated seizures (42, 43). Telfeian et al. observed that the smaller the size of glioblastoma multiforme, the higher the risk of postoperative seizures. This could be because more brain tissue dissection may be required to reach smaller tumors, thus explaining the higher risk of seizures after surgery for smaller tumors (44). In patients with partially resected gliomas, the residual tumor may continue to invade brain tissues and stimulate the cerebral cortex, leading to abnormal cortical discharges and triggering seizures (45). Therefore, the patient's condition should be considered during clinical treatment, and the tumor should be removed as completely as possible. If complete removal of the tumor is not possible, early prevention and treatment of seizures should be actively pursued before and after surgery to decrease the risk of early postoperative seizures and improve prognosis.

The current study has the following limitations: First, the number of articles included in this review was small, and most of these articles were from China, which may be subject to selection bias. Second, the diagnostic criteria for glioma and seizures used in the included studies were not consistent, which may account for significant heterogeneity. Third, the analysis process of this study did not distinguish between odds ratio (OR), relative risk (RR), or hazard ratio (HR), and although the actual difference between the three is not significant, there is some difference in the risk assessments of the diseases they essentially measure, which may lead to some bias in the results.

## 6 Conclusion

Based on the available evidence, history of seizures, dyskinesia, frontal lobe tumors, pathological grade ≤ 2, tumor ≥3 cm, partial tumor resection, peritumor edema ≥2 cm, and glioma cavity hemorrhage are identified as risk factors for early postoperative seizures, and clinical practitioners can combine these indicators for early detection, diagnosis, and intervention in such kind of cases, thereby improving the quality of life of affected individuals.

## Data availability statement

The original contributions presented in the study are included in the article/Supplementary material, further inquiries can be directed to the corresponding author.

## Author contributions

BS: Conceptualization, Data curation, Formal analysis, Funding acquisition, Investigation, Methodology, Project administration, Resources, Software, Supervision, Validation, Visualization, Writing – original draft, Writing – review & editing. YS: Conceptualization, Data curation, Formal analysis, Funding acquisition, Investigation, Methodology, Project administration, Resources, Software, Supervision, Validation, Visualization, Writing – original draft, Writing – review & editing. ZW: Conceptualization, Data curation, Formal analysis, Investigation, Methodology, Project administration, Software, Writing – original draft. CZ: Investigation, Methodology, Software, Writing – original draft. LY: Conceptualization, Data curation, Formal analysis, Funding acquisition, Investigation, Methodology, Project administration, Resources, Software, Supervision, Validation, Visualization, Writing – review & editing.

## Supplementary material

Supplementary Material 1 Search history.

Supplementary Table S1 Literature characteristics.

Supplementary Table S2 NOS scores.

Supplementary Table S3 Single-factor meta-analysis.

Supplementary Table S4 Multi-factor meta-analysis.

Supplementary Figure S1 **(A)** Meta-analysis of the prevalence of glioma complicated with early postoperative seizures; **(B)** Sensitivity analysis of the prevalence of glioma with early postoperative seizures; **(C)** Egger's test of the prevalence of glioma complicated with early postoperative seizures.
