# Supplementary material for: Prevalence and risk factors of early postoperative seizures in patients with glioma: a systematic review and meta-analysis
Source: Front Neurol. 2024 Mar 20;15:1356715. doi: 10.3389/fneur.2024.1356715 (PMC10989274; doi:10.3389/fneur.2024.1356715)
Supplement: Supplementary Table S1 — Literature characteristics. [file Table_1.docx]

Table S1 Table of literature characteristics

| study | country | study design | sample size | | Gender(M/F) | Low grade glioma/ High-grade glioma | | Age(years) | |
| --- | --- | --- | --- | --- | --- | --- | --- | --- | --- |
|  |  |  | S | NS |  | S | NS | S | NS |
| YD Dong 2019 | China | case control | 22 | 78 | 58/42 | 15/7 | 13/65 | 51.2 | 50 |
| H Deng 2022 | China | case control | 15 | 67 | 31/51 | 5/31 | 10/36 | 48.58 | |
| XB Gui 2012 | China | case control | 16 | 240 | 161/95 | 9/122 | 7/118 | 34.5 | |
| Hasmujiang 2017 | China | case control | 49 | 183 | 81/151 | NA | | 54.7 | |
| JT Hu 2014 | China | case control | 23 | 247 | 150/120 | 13/130 | 10/115 | 37.68 | |
| C Tian 2022 | China | case control | 39 | 124 | 98/65 | 15/66 | 24/77 | 7-81 | |
| SW Yang 2014 | China | case control | 37 | 136 | 101/72 | 18/27 | 19/109 | 21-71 | |
| P Zhang 2014 | China | case control | 28 | 136 | 94/70 | 15/29 | 13/107 | 19-37 | |
| XC Zhang 2021 | China | case control | 55 | 267 | 188/134 | 36/53 | 19/214 | 52.13 | 51.07 |
| Yuen 2012 | Australia | case control | 73 | 117 | 105/85 | 22/51 | 16/101 | 45.9 | 57.9 |
| Yu 2019 | China | case control | 131 | 456 | 371/216 | NA | | 18-80 | |

S: Glioma with epilepsy; NS: Glioma without epilepsy; NA: Not applicable

Table S2 NOS scores

| Study | Is the case definition adequate? | Representativeness of the cases | Definition of Controls | Comparability of cases and controls based on the design or analysis | Ascertainment of exposure | Same method of ascertainment for cases and controls | Non response | Total scores |
| --- | --- | --- | --- | --- | --- | --- | --- | --- |
| YD Dong 2019 | * | * | * | ** | * | * | * | 8 |
| H Deng 2022 | * | * | * | * | * | * | - | 6 |
| XB Gui 2012 | * | * | * | * | * | * | * | 7 |
| Hasmujiang 2017 | * | * | * | ** | * | * | * | 8 |
| JT Hu 2014 | * | * | * | * | * | * | * | 7 |
| C Tian 2022 | * | * | * | * | * | * | * | 7 |
| SW Yang 2014 | * | * | * | ** | * | * | * | 8 |
| P Zhang 2014 | * | * | * | ** | * | * | * | 8 |
| Lettieri 2023 | * | * | * | ** | * | * | * | 8 |
| Yuen 2012 | * | * | * | ** | * | * | * | 8 |
| Yu 2019 | * | * | * | ** | * | * | * | 8 |

Table S3 Single factor meta-analysis

| Risk factors | No of study | heterogeneity | | RR (95%CI) | P | Egger |
| --- | --- | --- | --- | --- | --- | --- |
|  |  | I^2^(%) | P |  |  |  |
| Male | 11^[22-32]^ | 2.7 | 0.416 | 1.10 (1, 1.22) | 0.06 | 0.876 |
| Female | 11^[22-32]^ | 0 | 0.465 | 0.90 (0.79, 1.01) | 0.07 | 0.520 |
| Epilepsy history | 9^[22-30]^ | 24.6 | 0.225 | 1.94 (1.76, 2.14) | 0.001 | 0.137 |
| Dyskinesia | 3^[22,25,30]^ | 89.2 | 0.0001 | 3.13 (1.20, 8.15) | 0.02 | 0.235 |
| Circulatory disease | 3^[22,25,30]^ | 0 | 0.878 | 0.88 (0.67, 1.16) | 0.365 | 0.567 |
| Metabolic disease | 3^[22,25,30]^ | 53 | 0.119 | 1.05 (0.59, 1.86) | 0.876 | 0.819 |
| Frontal lobe tumor | 9^[22-24,26-31]^ | 75.6 | 0.0001 | 1.45 (1.16, 1.83) | 0.001 | 0.421 |
| Pathological grade <2 | 8^[22-24,26-30]^ | 85.9 | 0.0001 | 1.74 (1.13, 2.67) | 0.012 | 0.452 |
| Tumor >3cm | 8^[22-24,26-30]^ | 92.4 | 0.0001 | 1.70 (1.18, 2.45) | 0.005 | 0.148 |
| Tumor boundary blurring | 3^[22,25,30]^ | 27.3 | 0.253 | 1.07 (0.93, 1.23) | 0.355 | 0.451 |
| Partial tumor resection | 7^[22-24,26-29]^ | 7.7 | 0.369 | 1.60 (1.36, 1.88) | 0.001 | 0.062 |
| Peritumoral edema >2cm | 6^[22-23,25,27-29]^ | 63.7 | 0.017 | 1.77 (1.40, 2.25) | 0.001 | 0.133 |
| Lumen hemorrhage | 6^[22-23,25,27-29]^ | 83.9 | 0.001 | 3.15 (1.85, 5.37) | 0.001 | 0.202 |
| Prophylactic drug | 3^[22,27,30]^ | 97 | 0.001 | 2.40 (0.41, 14.23) | 0.33 | 0.081 |

Table S4 Multi-factor meta-analysis

| Risk factors | No of study | heterogeneity | | ES (95%CI) | P | Egger |
| --- | --- | --- | --- | --- | --- | --- |
|  |  | I^2^(%) | P |  |  |  |
| Epilepsy history | 9^[22-30]^ | 84.7 | 0.0001 | 2.54 (1.24, 5.20) | 0.011 | 0.411 |
| Dyskinesia | 3^[22,25,30]^ | 48.1 | 0.146 | 2.53 (1.83, 3.51) | 0.001 | 0.031 |
| Frontal lobe tumor | 5^[22-26]^ | 89 | 0.0001 | 2.45 (0.92, 6.55) | 0.073 | 0.17 |
| Tumor >3cm | 4^[22,28-30]^ | 0 | 0.874 | 2.56 (1.99, 3.31) | 0.001 | 0.667 |
| Partial tumor resection | 3^[22-23,28]^ | 56.3 | 0.102 | 1.36 (0.88, 2.11) | 0.172 | 0.534 |
| Peritumoral edema >2cm | 6^[22-23,25,27-29]^ | 33.7 | 0.183 | 2.40 (1.90, 3.03) | 0.001 | 0.303 |
| Lumen hemorrhage | 3^[24,26-27]^ | 0 | 0.847 | 2.93 (1.79, 4.81) | 0.001 | 0.124 |
| Prophylactic drug | 4^[22,25,27,30]^ | 94.7 | 0.001 | 0.79 (0.15, 4.05) | 0.778 | 0.336 |
