# Supplementary material for: Prevalence and risk factors of early postoperative seizures in patients with glioma: a systematic review and meta-analysis
Source: Front Neurol. 2024 Mar 20;15:1356715. doi: 10.3389/fneur.2024.1356715 (PMC10989274; doi:10.3389/fneur.2024.1356715)
Supplement: Supplementary Figure S1 — (A) Meta-analysis of the prevalence of glioma complicated with early postoperative seizures; (B) Sensitivity analysis of the prevalence of glioma with early postoperative seizures; (C) Egger's test of the prevalence of glioma complicated with early postoperative seizures. [file Data_Sheet_2.docx]

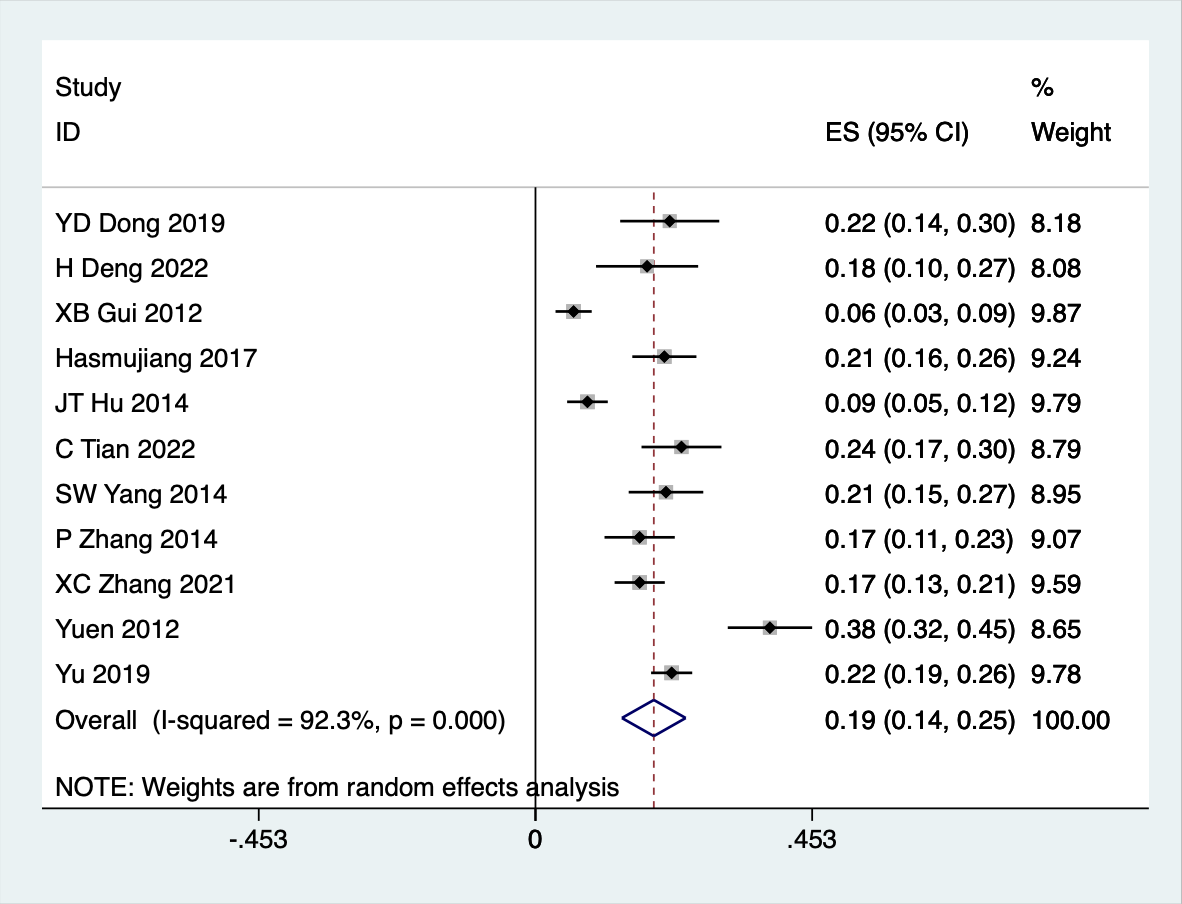


A: Meta-analysis of the prevalence of glioma complicated with early postoperative seizures.


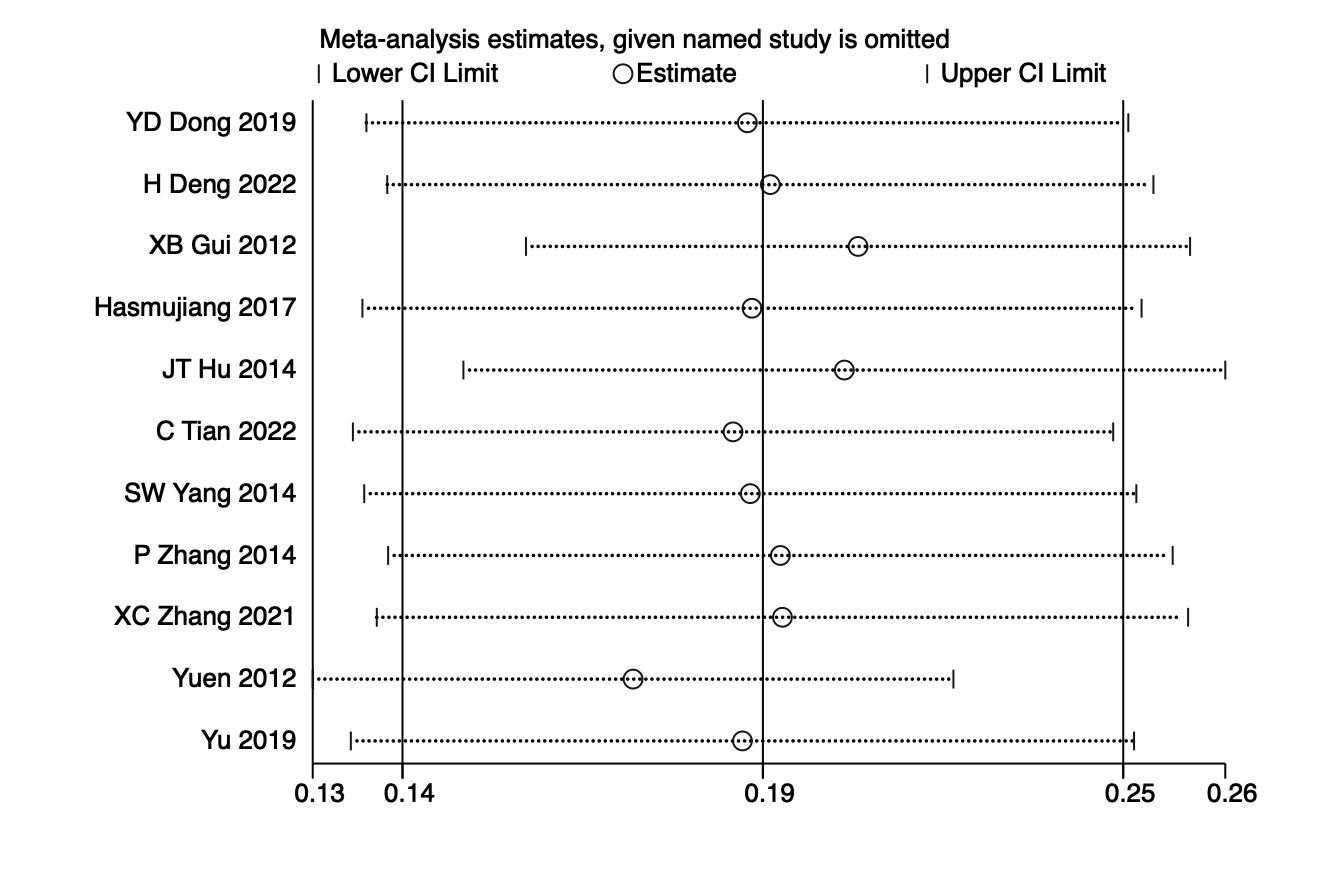


B Sensitivity analysis of the prevalence of glioma with early postoperative seizures.


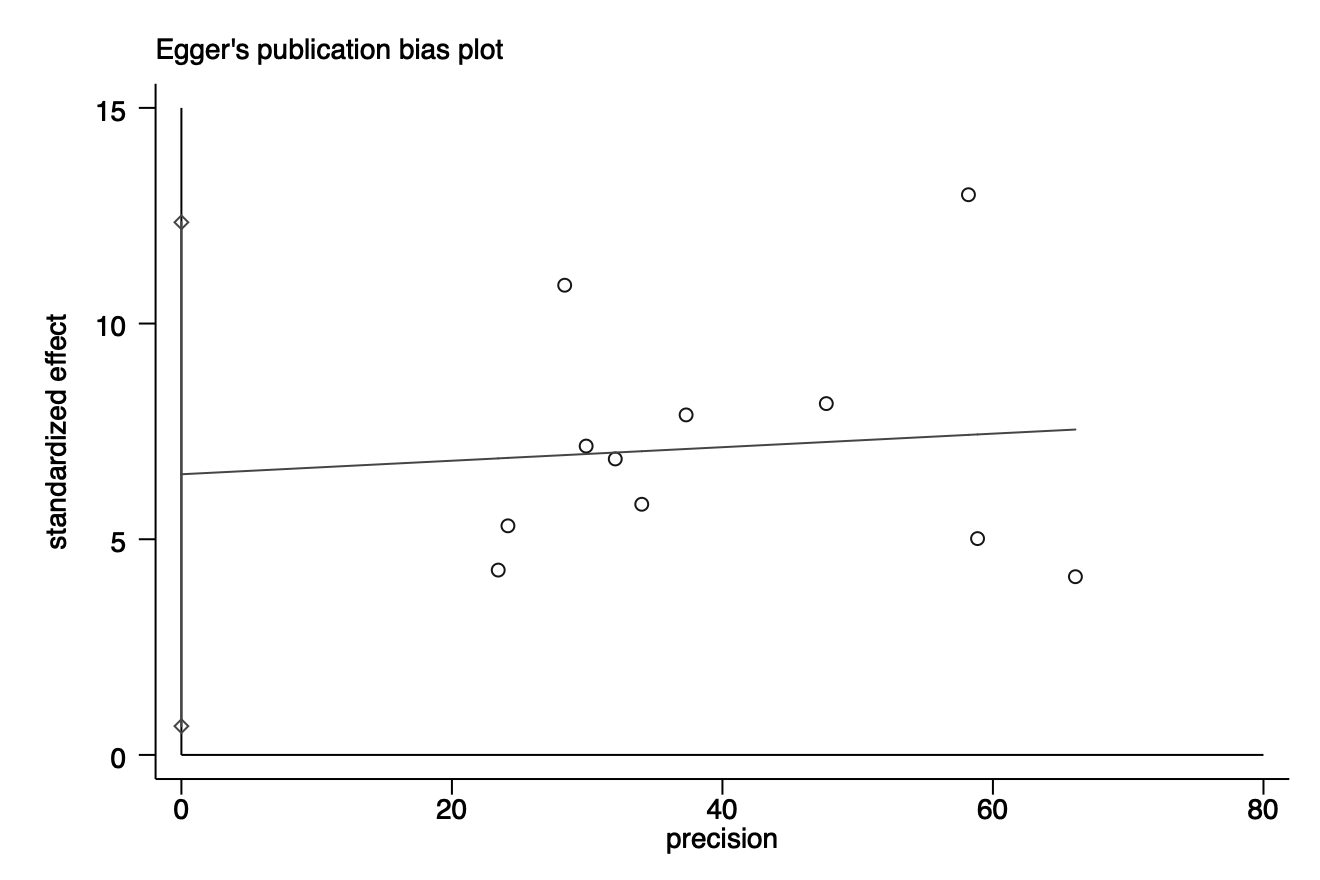


C: Egger test of the prevalence of glioma complicated with early postoperative seizures.
